# Enhanced terahertz detection of multigate graphene nanostructures

**DOI:** 10.1515/nanoph-2021-0573

**Published:** 2022-01-04

**Authors:** Juan A. Delgado-Notario, Wojciech Knap, Vito Clericò, Juan Salvador-Sánchez, Jaime Calvo-Gallego, Takashi Taniguchi, Kenji Watanabe, Taiichi Otsuji, Vyacheslav V. Popov, Denis V. Fateev, Enrique Diez, Jesús E. Velázquez-Pérez, Yahya M. Meziani

**Affiliations:** CENTERA Laboratories, Institute of High Pressure Physics, Polish Academy of Sciences, 29/37 Sokołowska Str, Warsaw, Poland; Nanotechnology Group, USAL-Nanolab, Universidad de Salamanca, Plaza de la Merced, Edificio Trilingüe, 37008, Salamanca, Spain; International Center for Materials Nanoarchitectonics, National Institute for Materials Science, 1-1 Namiki, Tsukuba 305-0044, Japan; Research Center for Functional Materials, National Institute for Materials Science, 1-1 Namiki, Tsukuba 305-0044, Japan; Research Institute of Electrical Communication, Tohoku University, Sendai 980-8577, Japan; Kotelnikov Institute of Radio Engineering and Electronics (Saratov Branch), Russian Academy of Sciences, Saratov 410019, Russia

**Keywords:** 2D materials, field effect transistor, graphene, nano-photodetector, plasmonics, terahertz

## Abstract

Terahertz (THz) waves have revealed a great potential for use in various fields and for a wide range of challenging applications. High-performance detectors are, however, vital for exploitation of THz technology. Graphene plasmonic THz detectors have proven to be promising optoelectronic devices, but improving their performance is still necessary. In this work, an asymmetric-dual-grating-gate graphene-terahertz-field-effect-transistor with a graphite back-gate was fabricated and characterized under illumination of 0.3 THz radiation in the temperature range from 4.5 K up to the room temperature. The device was fabricated as a sub-THz detector using a heterostructure of h-BN/Graphene/h-BN/Graphite to make a transistor with a double asymmetric-grating-top-gate and a continuous graphite back-gate. By biasing the metallic top-gates and the graphite back-gate, abrupt *n^+^n* (or *p^+^p*) or *np* (or *pn*) junctions with different potential barriers are formed along the graphene layer leading to enhancement of the THz rectified signal by about an order of magnitude. The plasmonic rectification for graphene containing *np* junctions is interpreted as due to the plasmonic electron-hole ratchet mechanism, whereas, for graphene with *n^+^n* junctions, rectification is attributed to the differential plasmonic drag effect. This work shows a new way of responsivity enhancement and paves the way towards new record performances of graphene THz nano-photodetectors.

## 1 Introduction

Over the last decades, development of terahertz (THz) devices in the so-called “THz-GAP” (0.1–10 THz) has been one of the hottest topics in modern physics due to a potential use of the THz radiation in various application areas such as security [1], material sensing [2], wireless communication [3] and others [4, 5]. Graphene has become a promising material for detecting THz radiation due to its unique opto-electronic properties [6]. For example, graphene exhibits high carrier mobility, fast carrier dynamics resulting in a high optical response speed. It also demonstrates a strong light–matter interaction that is related to the excitation of plasmons (i.e., collective oscillations of charge carries) whereby studies of THz rectification by graphene structures have led to the development of novel plasmonic graphene-terahertz-field-effect-transistors (GTeraFETs) [7], [8], [9], [10], [11]. In plasmonic TeraFET structures, the penetration depth of THz radiation is characterized by the so-called diffusion length that is a direct function of carrier mobility and ranges from tens of nanometers in silicon CMOS to few hundred nanometers in graphene [12, 13]. The region of the FET channel that is longer than this diffusion length is inactive and deteriorates the FET performance because it adds a load resistance, increasing the detector noise and decreasing the effective rectification signal. Therefore, plasmonic TeraFETs have dimensions in the nanometer range for an efficient detection of THz radiation. Moreover, for almost any THz detection mechanisms some asymmetric designs of the plasmonic TeraFETs are required. Several strategies are used to introduce asymmetries into the plasmonic THz devices, such as different types of metallization of the electrical contacts [14], special design of coupled antennas [15], small drain-to-source dc currents [16] and voltages [13] as well as asymmetric double grating-gate periodic nanostructures (ADGGS). GTeraFETs with ADGGS have been demonstrated to be an outstanding solution because they strengthen the coupling of incoming THz radiation to the graphene channel, leading to an enhancement of the plasmons excitations [17]. Different ADGGS nanostructures were reported, establishing a new generation of the plasmonic GTeraFETs for detection [18], emission [19] or amplification [20] of THz radiation.

Plasmonic rectification of THz radiation in asymmetric multiple-gate periodic nanostructures has been comprehensively studied theoretically, where the interaction between incoming THz radiation and graphene plasmons occurs efficiently due to the strong coupling and a relatively low level of losses [21]. Two physical mechanisms are postulated to contribute with comparable strength to the plasmonic rectification of THz radiation in these nanostructures: plasmonic drag of charge carriers and plasmonic electron-hole ratchet [22]. Recently, it has been theoretically predicted that plasmonic THz rectification in a spatially inhomogeneous graphene channel could be significantly enhanced close to the Dirac point of graphene [23]. This theoretical prediction has not been experimentally verified so far.

In this work we investigate sub-THz radiation response by using an asymmetric dual-grating-gate GTeraFET (ADGG-GTeraFET) with an additional graphite back-gate. The graphite back-gate screens the charge inhomogeneities induced by the SiO_2_ surface and, what is more important, ensures a perfect control of the carrier concentration along the graphene channel. The device was characterized under illumination of 0.3 THz radiation in the temperature range between 4.5 K and room temperature. A significant enhancement of the rectified THz signal was observed when abrupt potential barriers along the graphene were created by suitably biasing the two metallic top gates and the graphite back-gate. The enhancement factor of up to 6 at 4.5 K and 2 at room temperature was observed when the back-gate and top-gate biasing allowed for the creation of spatial inhomogeneities in graphene layer, giving rise to the establishment of multiple *pn (np)* or *n^+^n (p^+^p)* junctions and therefore inducing multiple abrupt potential barriers along the graphene sheet. In this work we show that using the continuous back gate and asymmetric top double-grating gate one can create multiple junctions and hence enhance the detector responsivity by about an order of magnitude.

### 1.1 Graphene TeraFET detector

**Figure 1:**
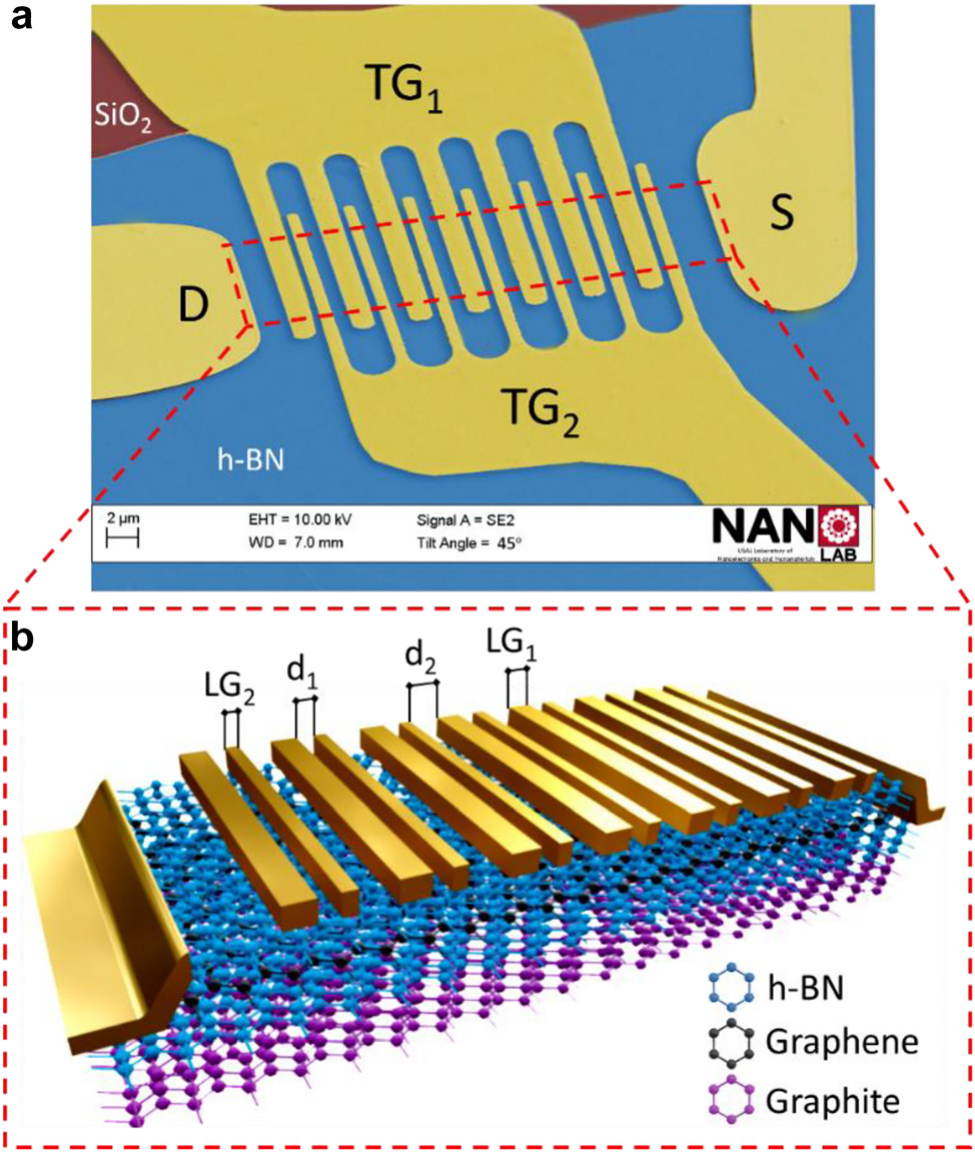
ADGG graphene-based THz detector. (a) False colour SEM tilted image of the fabricated ADGG-GTeraFET where drain (D), source (S), top gate 1 (TG_1_), top gate 2 (TG_2_) terminals are labeled. Top h-BN and SiO_2_ are colored in blue and garnet respectively and the red dashed line highlights the edges of the graphene sheet to guide the eye. (b) 3D schematic view (not to scale) of the highlighted red dashed area showing the structure of the fabricated ADGG-GTeraFET consisting of two asymmetrically situated top gates, a monolayer graphene sheet encapsulated between two flakes of hexagonal boron nitride and a few-layers thick graphite to be used as the back-gate. Drain and source were fabricated as one-dimensional edge contacts with the graphene sheet.

A graphene TeraFET with two asymmetrically situated top metallic gates and a few-layers thick graphite back gate was designed and fabricated for use as a photodetector of THz radiation (Figure 1(a)). The core nanostructure of the fabricated device consisted of a graphene-based double heterostructure, hBN/Graphene/hBN/Graphite, fabricated by a dry transfer technique [24] and transferred onto a commercial Si/SiO_2_ substrate. The graphene active region of the transistor was encapsulated between two relatively thick dielectric h-BN flakes (∼28 nm for the top and ∼50 nm for the bottom one) and graphite back-gate that additionally screened remote charge impurities trapped in the SiO_2_ substrate and could improve the quality of the final device [25]. h-BN was used as substrate dielectric insulator and, at the same time, as the neutral passivation layer to encapsulate the graphene sheet, protecting it from ambient atmosphere (which leads to device degradation with time if not encapsulated) [26, 27]. Drain and source were fabricated as side contacts by using electron beam lithography (EBL) and then the heterostructure was dry-etched in an ICP-RIE Plasma Pro Cobra 100 with a SF_6_ atmosphere (40 SCCM, *P* = 6 mTorr, *P* = 75 W at 10 °C) and subsequently 3.5 nm of Cr and 60 nm of Au were deposited by the electron beam evaporation. Sides of the etched structure have pyramidal profiles and thus the contacts between the “exposed” graphene channel and the drain and source metallization are improved [28]. Finally, the top gates as well as the contact for the back-gate graphite were fabricated by a second round of EBL followed by electron beam evaporation of Cr/Au (5/45 nm thick). Figure 1(b) shows a 3D cross-sectional view of the full device along with its geometrical parameters (for a unit cell the dimensions are the following: LG_1_ = 1500 nm, LG_2_ = 750 nm, *d*_1_ = 500 nm, *d*_2_ = 1000 nm). The top h-BN flake is used as an insulator for the top gates (TGs) and the bottom h-BN flake insulates the graphene channel from the graphite back gate (BG). The total channel length is 31 μm. (More information can be found in the Supplementary Materials 1).

## 2 Results and discussion

### 2.1 Transport measurements

The device was placed inside a variable temperature pulse-tube cryostat with an optical access through a polyethylene window transparent to THz radiation. Prior to THz photoresponse measurements, the device was electrically characterized in DC within the temperature range from 4.5 K to room temperature. The 2-terminal channel resistance of the ADGG-GTeraFET between drain and source was measured by using a standard lock-in technique where a pseudo-dc current (11 Hz) of 10 nA was injected into the drain and then collected in the Source while the voltage drop between the drain and source contacts was measured by a lock-in amplifier (SR860).

**Figure 2:**
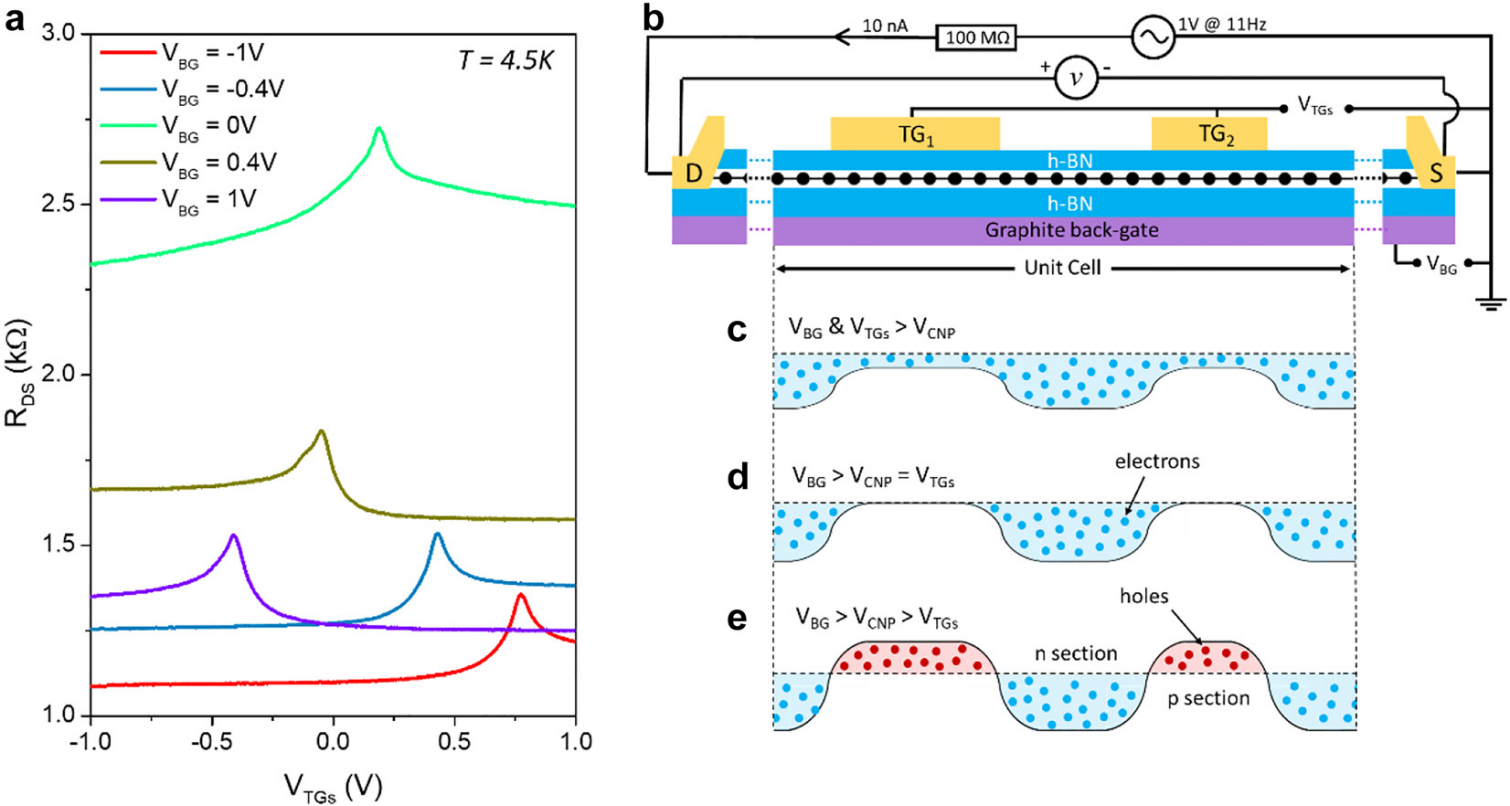
Electrical characteristics of the ADGG-GTeraFET. (a) Two-terminal resistance (R_DS_) of the ADGG-GTeraFET as a function of the top gates (TG_1_ and TG_2_) bias voltage for different back-gate voltages at *T* = 4.5 K. (b) Schematic electrical circuit of the ADGG-GTeraFET for the transport measurements, where a 10 nA pseudo-dc current at 11 Hz, generated by an AC voltage source in series with a 100 MΩ resistance, was injected into the drain contact (current flow is indicated by the arrow) and the voltage drop (*v*) was measured between drain and source contacts. Only the asymmetric unit cell was included for simplification. (c)–(e) Energy-band diagrams of the graphene channel in the asymmetric unit cell showing the charge distributions at zero drain voltage for the different biasing conditions.

Figure 2(a) shows the drain-to-source resistance (*R*_DS_) as a function of the DC voltage applied to both TGs for different values of DC voltage applied to the back-gate (BG) at 4.5 K. The top gates (TG_1_ and TG_2_) are electrically connected to each other as shown in Figure 2(b). When the value of the BG bias voltage varies, the charge neutrality point (CNP) shifts and, therefore, the base line of *R*_DS_ also changes. This behaviour has been previously reported in dual-gated GFETs [29] where the shift of the CNP occurs if a positive (or negative) BG voltage is applied to the device and a specific level of concentration of electrons or holes is induced in the graphene sheet. Consequently, a voltage of the opposite sign needs to be applied to the TGs to reach a new CNP in the dual-gated areas of the graphene channel. The changes in the base line of the resistance (vertical offset) are caused by the resistance changes in the single-gated areas of graphene.

Furthermore, unlike the single-gated GFETs in which the channel resistance shows a symmetrical bell shape [30, 31], the multi-gated GFET exhibits clear asymmetric profiles in the channel resistance curves (Figure 2(a)) that may arise due to interband tunneling between the dual-gated and single-gated regions [32, 33]. The same behaviour was also observed in the device when DC transport measurements were performed at 77 K, and room temperature (See Supplementary Materials 2). Figure 2(c)–(e) show the energy-band diagrams in an unit cell of the structure under zero drain-to-source bias and for different biasing voltages at the top and the back gates. Depending on the combination of these voltages, different profiles of potential barriers could be created along the graphene channel, where the carrier type in different single and dual gated regions becomes either n and n^+^ (Figure 2(c)) or n^+^ and i (intrinsic) (Figure 2(d)). Here the superscript “+” indicates regions having amount of majority charge carriers higher by about one order of magnitude than the ones without the superscript. Also, abrupt n–p junctions (Figure 2(e)) can be formed along the graphene channel when the gates are suitably biased. As we will show hereunder, the behaviour of the rectified signal generated by the incoming 0.3 THz radiation is strongly affected by these bias conditions.

Additionally, various key parameters of the transistor such as the parasitic resistance (*R*_o_) of the transistor (i.e., the sum of the contact resistances and the resistance of the un-gated regions of the device), carrier mobilities (*μ*_e_ for electrons or *μ*_h_ for holes) as well as carrier concentrations (electrons or holes) along the different parts of the graphene channel were obtained by fitting the measured data to the theoretical model presented in Refs. [34, 35]. Table 1 shows the obtained mobility (*μ*_e_ & *μ*_h_) at 4 K and room temperature showing the excellent quality of the graphene channel of the ADGG-GTeraFET. More information can be found in Supplementary Materials 3.

**Table 1:** Electron and hole mobilities in graphene channel of the ADGG-GTeraFET.

Temperature	Electron mobility, *µ*_e_	Hole mobility, *µ*_h_
(K)	(cm^2^/V s)	(cm^2^/V s)
4.5 K	78,500	74,000
300 K	38,000	32,000

### 2.2 Terahertz measurements

To perform the THz photoresponse measurements, a THz solid-state harmonic generator source fabricated by RPG Radiometer Physics GmbH, with a customized design based on a dielectric resonator oscillator (DRO) with an initial frequency of 12.5 GHz and Schottky diode multiplier stages, was used to generate a continuous wave radiation source operating at 0.3 THz (multiplication factor of 24) with an output power of about 6 mW. The power emitted by the THz source was measured close to the output of the source using a calibrated pyroelectric detector. The output THz radiation was firstly modulated by using a mechanical chopper at 333 Hz, collimated with an off-axis gold parabolic mirror and finally focused on the ADGG-GTeraFET by using a THz lens made of TPX (Polymethylpentene) with a focal distance of 100 mm. The photocurrent generated by THz radiation was measured in the drain contact by using a low noise current preamplifier (Stanford Research, SR570) with a sensitivity of 1 μA/V in series with a lock-in amplifier, while the Source was kept grounded. Drain contact was kept unbiased during all THz detection measurements. More information can be found in Supplementary Materials 4.

**Figure 3:**
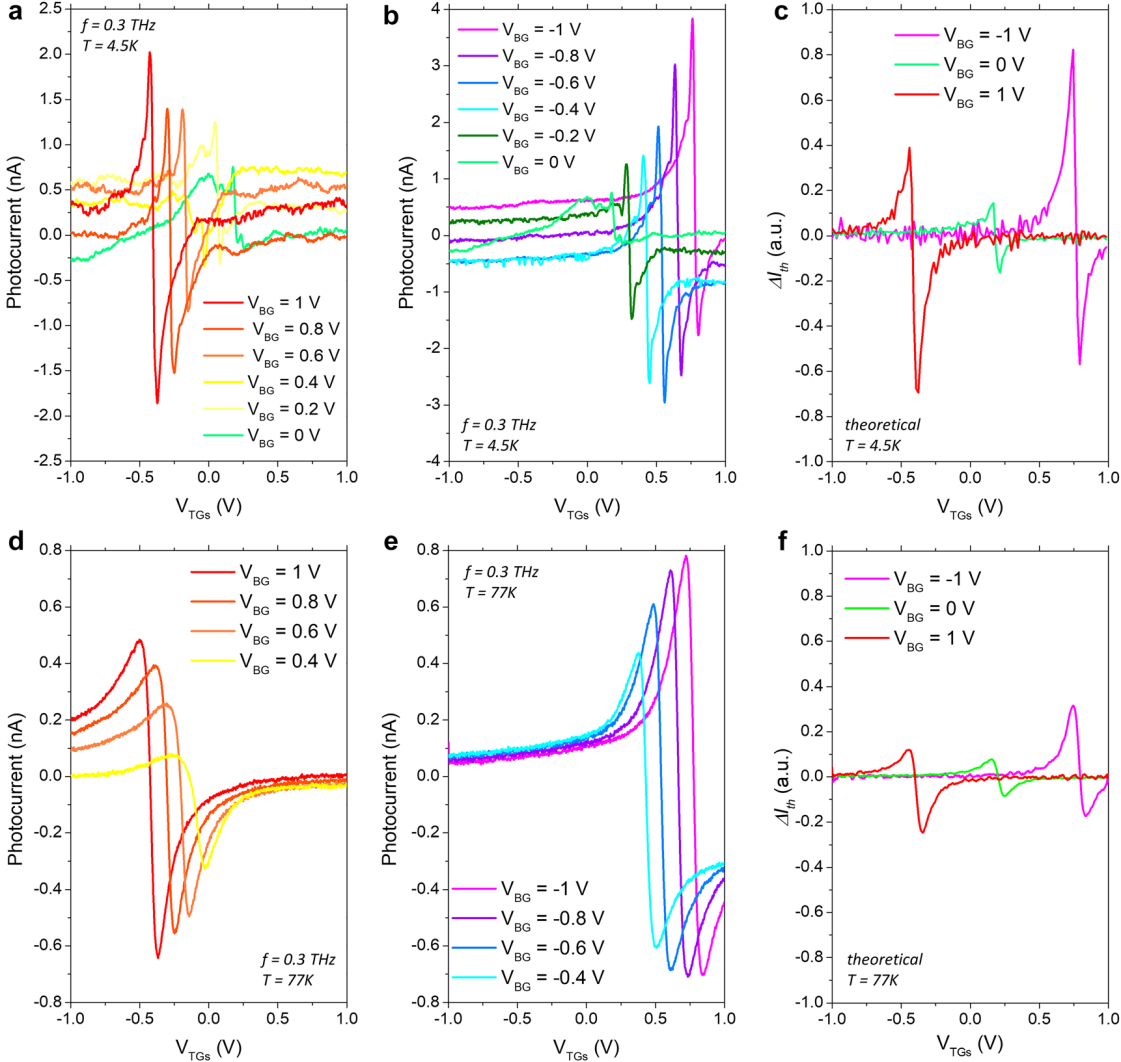
THz photodetection enhancement. Experimental photocurrent as a function of the TGs voltage for different (a) positive and (b) negative BG bias at 0.3 THz. (c) The current photoresponse (*ΔI*_th_) predicted by the analytical model as a function of the TGs bias voltage for three different values of the BG bias voltage at *T* = 4.5 K. Experimental photocurrent as a function of the TGs voltage for (d) positive and (e) negative BG voltages at 0.3 THz. (f) The expected theoretical value of the current photoresponse as a function of the TG bias voltage at 77 K.

Figure 3 shows the measured photocurrent as a function of the TGs voltage for different positive (a) and negative (b) values of the BG voltage at 4.5 K. At *V*_BG_ = 0 V, the photocurrent shows a maximum value of 0.75 nA when the device operates close to the CNP. The signal increases as the graphene channel nears the CNP and when the CNP is reached, the photoresponse abruptly changes its sign due to the change of the charge carrier type which is consistent with the ambipolar nature of carrier transport in graphene. The photoresponse exhibits a behaviour similar to the one reported in previous works on graphene-based THz detectors [7], [8], [9, 15, 36, 37]. Various mechanisms of the THz radiation photocurrent generation were used for interpretation, like plasmonic rectification [7], photo-thermoelectric (PTE) effect (PTE) [36, 38] and plasmonic or electronic ratchet effects [39, 40]. To explore the interplay of different effects that result in conversion of the THz wave in a DC current, it is necessary to perform multiple studies on; polarization and frequency dependencies, photocurrent rectification with gates bias, variation of the structure geometry and/or different electronic properties such as various relaxation times as shown in Refs. [39, 40]. Following results of these works that have been performed on very similar architecture nanostructures: monolayer [39] and bilayer [40] graphene, we attribute the observed THz photocurrent signals as due to plasmonic rectification ratchet effects. Theoretical description of such effects is also given in Ref. [22].

When positive voltages are applied to the graphite BG, the graphene sheet is uniformly n-doped, and the TGs have to be negatively biased to reach a new CNP, leading to an inhomogeneous graphene channel. Varying the bias voltage applied to the TGs induces different abrupt potential barriers due to the n^+^n and np junctions formed through the graphene channel. According to Ref. [22] one can distinguish two cases: for graphene containing np junctions, the plasmonic rectification can be attributed mainly to the plasmonic electron–hole ratchet mechanism, while for graphene with n^+^n junctions, rectification is due to the differential plasmonic drag effect. Independently, we found experimentally that the measured photoresponse generated by the incoming THz radiation can be substantially and systematically enhanced when different potential barriers are created at larger BG and TGs bias voltages. The measured signal reached a maximum value of 2 nA close to the CNP at *V*_BG_ = 1 V (Figure 3(a)). Likewise, we found a similar behaviour when negative voltages were applied to the graphite BG. A maximum signal of approximately 4 nA at *V*_BG_ = –1 V was obtained while biasing the TGs (Figure 3(b)). These observations provide an argument in favor of the plasmonic rectification interpretation of the observed signals. In particular, the rise of the photoresponse with the increase of the barrier height and its maximum at the CNP are consistent with the theoretical prediction for the overdamped plasma-wave regime, which explain the signal enhancement phenomena by increasing of the plasmon near field and its inhomogeneity across a deeper barrier especially near the CNP [23].

We would like to stress, however, that in contrast to all earlier works on plasmonic ratchet effects, the specific design of our ADGG-GTeraFET that uses a dual grating-gate nanostructure and a graphite back-gate enables very significant channel non-uniformity and leads to a very important growth of the photoresponse by almost one order of magnitude.

To understand and analyze the behaviour of the GTeraFET as a THz detector, we used DC transport characteristics to calculate expected photocurrent in the so-called overdamped plasma-wave regime [8]. THz rectification takes place in this regime when the quality factor *Q* is such that Q=2πfτ<1 , where *f* is the frequency of the impinging radiation and *τ* is the carrier scattering time. In the TeraFET studied in the present work, the estimated value of the carrier scattering time is ∼ 400 fs and hence the quality factor value is ∼ 0.75. Therefore, the device operates in the overdamped plasma-wave regime and theoretically its voltage photoresponse is given by the phenomenological formula that relates DC characteristics with THz response [8]:

(1) $$\Delta U_{\mathrm{th}}\propto -\frac{1}{\sigma }\left(\frac{d\sigma }{dV_{\mathrm{GS}}}\right)$$

where Δ*U*_th_ is the theoretical expected photovoltage, *σ* is the drain-to-source conductance of the detector (which equals L/W/*R*_DS_ with *R*_DS_, as above introduced, the drain-to-source resistance), and *V*_GS_ is the gate-to-source DC voltage. Hence, the predicted photocurrent response could be deduced using the Ohm’s law [15]: Δ*I*_th_ = Δ*U*_th_/*R*_DS_.

Figure 3(c) shows Δ*I*_th_ for the ADGG-GFET at 4.5 K. When the BG is grounded, the theoretical current photoresponse follows the experimental photocurrent and reaches its maximum value when the graphene channel is close to the CNP. For higher values of the BG voltage, the behaviour of the expected current response is in good agreement with the experimental photoresponse. The theoretical current response increases for non-zero BG DC voltage, and the maximum response value is also obtained when the channel is close to a new CNP. Thus, the experimental enhancement of the photoresponse (Figure 3(a)–(b)) can be related to the changes of the channel resistance induced by the TGs and the BG bias voltages through the creation of multiple abrupt potential barriers along the graphene channel (Figure 2(c)–(e)). Also, interband tunneling between the channel’s dual- and single-gated regions may play a significant role when the gates are reversely biased [32, 33]. Indeed, for large negative BG voltages (*V*_BG_ ≤ −0.6 V), the photocurrent decreases at the n side (Figure 3(b)) whereby this degradation of the photoresponse can result from the multi-interband tunneling at larger negative BG voltages.

We also characterized the detector at higher temperatures and observed similar improvement trends in the experimental THz detection measurements and theoretical current photoresponse when applying higher BG voltages at 77 K (Figure 3(d)–(f)) and room temperature (See Supplementary Materials 5).

We would like to emphasize that the geometry of the device allows for the generation of different types of potential barriers along the graphene channel:

We concentrate the research on the so called “abrupt” lateral junctions that are created when the back gate and the metallic top gates create different density and/or type of carriers in the graphene channel. In the case under study, an “abrupt” lateral junction is expected to be created at the border between the dual-gated and the single-gated regions. The spread of such junction is determined by the density of carriers similar to standard p–n junction, where the extension of the space charge region depends on the doping levels of each side of the junction.

In contrast, the “smooth” lateral junctions are created by polarizing individually the neighbor top gates in the way that they have different density and/or type of carriers; meanwhile, the back-gate is not varied and keeps those single-gated regions at the charge neutrality point. In this case, different lateral junctions (n-i-n, p-i-p, p-i-n or n-i-p) are created, but they spread over a distance much larger than the “abrupt” ones.

While comparing to the “abrupt” ones, the “smooth” lateral junctions have shown different photocurrent behaviour as a function of the gate polarization (6-fold sign changing), similar to observed in Refs. [36], [37], [38]. This points towards the presence of photo-thermoelectric (PTE) effect in the “smooth” configuration.

In principle, the presence of the multiple p–n junctions in the “abrupt” configuration may also lead to PTE effect. However, we found that the gate voltage dependencies of the photocurrent in the “abrupt” configuration are very different from those observed in the “smooth” one: No six-fold sign changing, much stronger signal and strong enhancement of the signal with the back-gate bias. Moreover, the photocurrent depends on the applied DC source-to-drain voltage or current, what is in clear contradiction with the main argument of the PTE effect identification criteria of Ref. [36]. These and other arguments using results of Refs. [39, 40] allow us to attribute the photoresponse observed in the case “abrupt” junctions to the plasmonic ratchet effects.

For the sake of clarity, the results related to “smooth” junctions are presented only in the Supplementary Materials and the paper remains focused on the main observations/results of THz rectification enhancement by creation of “abrupt” lateral junctions.

**Figure 4:**
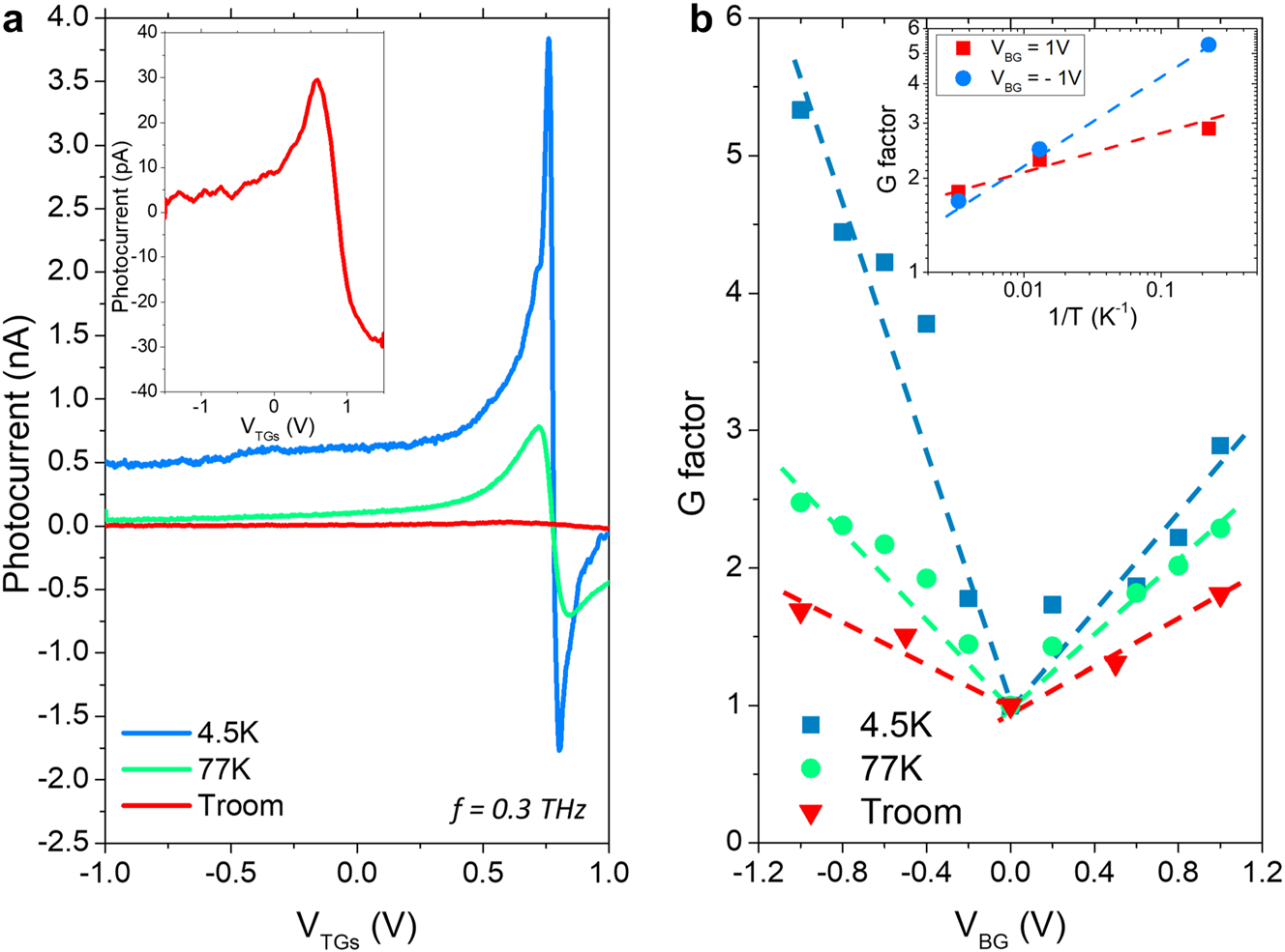
Temperature dependent THz photodetection. (a) Experimental photocurrent as a function of the TGs bias voltages at *V*_BG_ = –1 V for different temperatures (4.5 K, 77 K and room temperature). The top inset in panel (a) shows the photocurrent at room temperature. (b) Maximum experimental G factor (enhancement factor) as a function of the BG voltage at different temperatures. The top inset in panel (b) shows the G factor as a function of the temperature for two different values of BG bias, *V*_BG_ = –1 V and 1 V.

Additionally, though the predicted current response is generally in good agreement with the experimental results, the photosignal generated by the incoming THz radiation exhibits an offset at larger negative BG and TG voltages (*V*_BG_ = *V*_TGs_ = –1 V) that is not accounted for in the theoretical model which predicts that the signal should drop to zero. It was found that the value of this offset decreases with increasing temperature (Figure 4(a)). Following earlier works [41], we attribute the origin of this offset to the formation of additional p–n junctions at the boundaries of the channel (Drain and Source sides) between the n-type side contacts and the p-type graphene channel.

To quantify the results obtained for the photoresponse, the enhancement factor was calculated using the expression

(2) G=Signalmax(VBG≠0)Signalmax(VBG=0)

The *G* factor represents the ratio between maximum values of the measured photocurrent (Signal) for non-zero and zero BG voltages, respectively. If higher than unity, *G* factor measures the enhancement of the rectified signal associated to the back-gate voltage. Figure 4(b) shows the *G* factor as a function of the BG voltage at different temperatures. Figure 4(b) reveals that a clear enhancement of the rectified signal can be obtained by applying large BG voltages while biasing the TGs. A maximum *G* factor value close to 6 is obtained for *V*_BG_ = –1 V at 4.5 K. Similar behaviour is observed for all temperatures in the studied range. The *G* factor decreases when temperature increases but its value is still as large as 2 at room temperature. Moreover, at large BG voltages, the experimental *G* factor shows an exponential dependence on the temperature (see inset in Figure 4(b)). The considerable decrease of the rectified signal whit increasing temperature can be qualitatively understood as due to the blurring of the potential barrier with temperature that smooths the plasmon near-field distribution across the barrier. The quantitative description of this effect needs further theoretical consideration. Although we are able to explain qualitatively the main traces in the photocurrent versus gate voltage evolution using DC characteristics and phenomenological Eq. (1), we clearly need a more advanced microscopic physical model that could provide more quantitative description of the observed phenomena like for example: (i) the exponential dependence of the enhancement factor on the temperature and (ii) a close to linear dependence on the enhancement as a function of the BG voltage. At this point the experimental results presented above set a new challenge for theoretical research in THz plasmonics.

The current responsivity ( $R_{I}$ ), one of the most important figures of merit quantifying the photoresponse of the THz detector, was extracted from experimental THz measurements by the formula [42]

(3) $$R_{I}=\frac{\pi }{\sqrt{2}}\frac{\mathrm{Signal}}{P}\frac{S_{T}}{S_{D}}\text{,}$$

where the factor π2 comes from the Fourier transform of the modulated THz signal detected as its rms value is measured by the lock-in amplifier, *Signal* is the photocurrent measured (as defined above), *P* is the THz power at the graphene detector position (∼1 mW), $S_{T}$ is the THz beam spot area (∼7 mm^2^) and $S_{D}$ is the detector active area (∼31 × 10 μm^2^). The factor $S_{T}/S_{D}$ is the inverse coupling efficiency of the THz radiation to the active area of the GFET. It is worth noting that the coupling efficiency factor is introduced here to characterize the intrinsic performance of the plasmonic detector. This can be obtained under the perfect lossless coupling condition when the total power of the incoming THz radiation is focused into the active area of the detector with a larger active area [43], or using different techniques such as the integration of a perfectly matched antenna [38], or by optical elements such as aspheric focusing silicon lenses [7] and mesoscale dielectric particles [44].

Another figure of merit determining the performance of a photodetector is the noise-equivalent-power (NEP) given by the equation:

(4) $$\mathrm{NEP}=\frac{N}{R_{V}}=\frac{N}{R_{I}R_{\mathrm{DS}}}\text{,}$$

where *N* is the noise spectral density of the detector (in $V/\sqrt{\mathrm{Hz}}$ ) and *R*_V_ is the voltage responsivity. Since neither a bias voltage nor a bias current were applied during our THz measurements, the Johnson–Nyquist noise (or thermal noise) was the only source of noise that must be taken into account. Therefore, *N* is given by $N=\sqrt{4k_{B}TR_{\mathrm{DS}}}$ , where *k*_B_ is the Boltzmann constant and *T* is the temperature. The channel resistance *R*_DS_ was obtained from the transport measurements.

**Figure 5:**
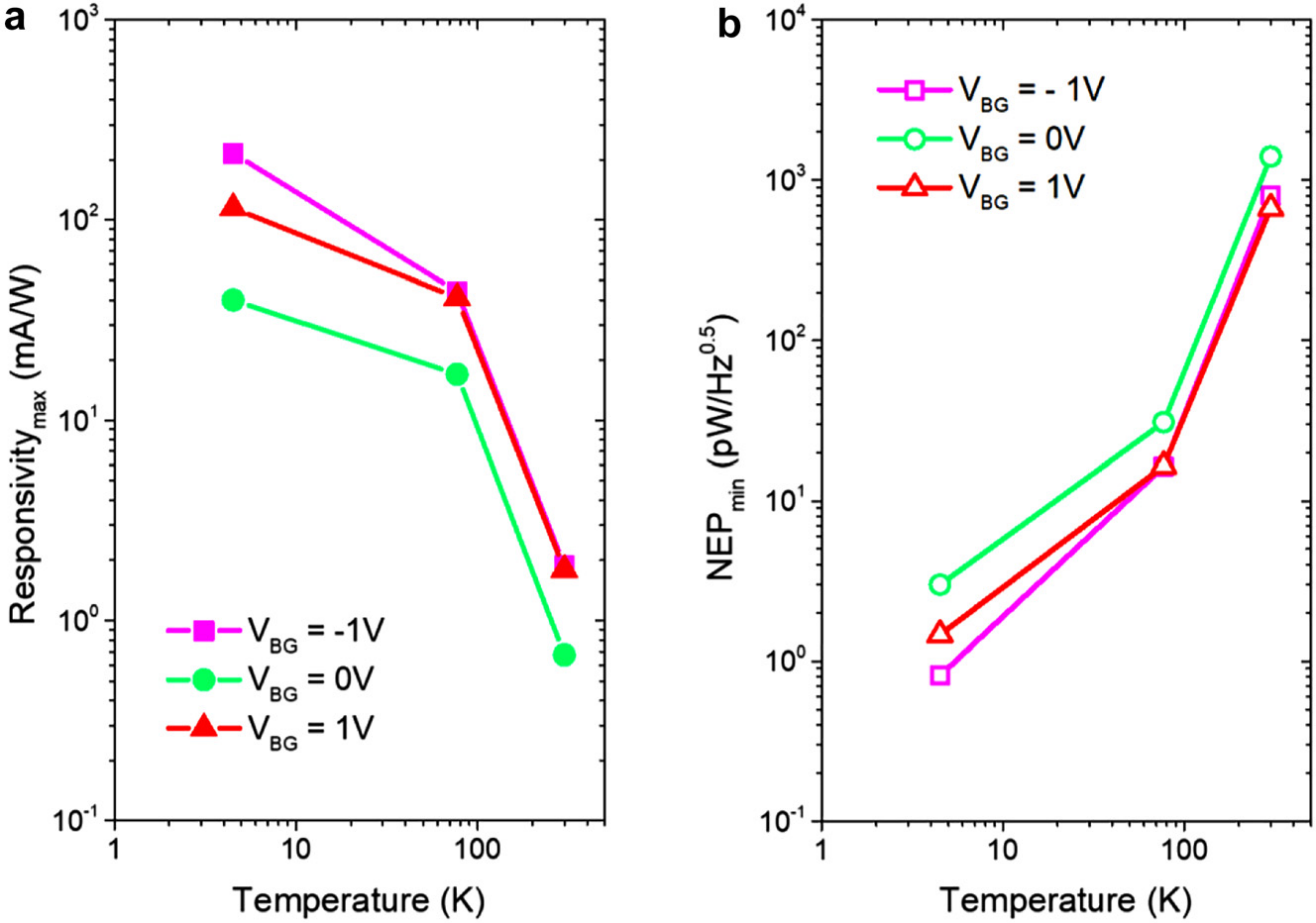
Performance of the ADGG-GTeraFET detector. Maximum current responsivity (a) and minimum NEP (b) values as a function of the temperature for three different BG voltages.

Figure 5 shows the maximum values of the current responsivity (a) and minimum NEP values (b) obtained for the ADGG-GTeraFET as a function of temperature. Maximum values of the current responsivity of 0.216 A/W and 1.9 mA/W were measured at 4.5 K and room temperature, respectively. The minimum values of the NEP of 0.81 pW/Hz^0.5^ and 0.67 nW/Hz^0.5^ were obtained at 4.5 K and room temperature, respectively. These values were obtained for a non-zero BG voltage. They are close to the values previously reported by other authors [8, 9, 15, 38] and they can still be improved by increasing the number of fingers of the gratings of the gates and improving the grating nanostructure geometry. As in many experimental cases, the graphene samples used in this study are much smaller than the THz wavelength [13]. Therefore, THz radiation is coupled to them through the metal pads and the bonding wires and the devices themselves work only as THz frequency rectifiers. The review of different coupling mechanisms and their influence on the THz responsivity of the plasmonic devices can be found in a recent work of Javadi et al. [45]. It is noteworthy that grating [46] or slit [47] structures have been recently studied to understand the influence of diffraction effects for THz detection and wireless communications. With regard to of the absolute performances of THz coupling, the device used in this work must be optimized, however this does not modify the studies/conclusions drawn about the role of multi-lateral junctions on the enhancement of the photoresponse of THz detectors. Certainly, the total responsivity and NEP of the transistor may be further improved using suitable lenses and antennas.

## 3 Conclusions

We have presented the experimental studies of THz radiation-induced photoresponse in the asymmetric-dual-grating-gate graphene-terahertz-field-effect-transistor in a wide temperature range (4.5–300 K). It is demonstrated experimentally that the rectified signal can be significantly enhanced when abrupt potential barriers along the graphene channel are formed by a proper biasing of the metallic top gates and the graphite back-gate. We have obtained values of the enhancement factor of the plasmonic rectification under 0.3 THz radiation close to 6 and 2 at 4.5 K and 300 K, respectively. Results of the measurements are in qualitative agreement with theoretical models of the plasmonic ratchet effect. However, there is yet no theory that quantitatively explains the enhancement that is linear with respect to gate voltage and exponential in function of temperature. Therefore, our work sets an important challenge for future theoretical work on THz plasmonics. Independently our work demonstrates the possibility of enhancing the responsivity and reducing the noise equivalent power of the plasmonic graphene-based THz photodetectors by means of an optimized design and a proper biasing of the device. Therefore, our work paves the way towards the design and fabrication of new graphene THz nano-photodetectors with record performances.

## Supplementary Material

Supplementary Material Details

## Supplementary Material

See Supplementary Material for additional information on the fabrication of the graphene based heterostructure, Raman characterization and processing of the ADGG-GTeraFET. Also, transport measurement results in the 4.5–300 K temperature range, experimental THz setup and THz measurements at frequency of 0.3 THz at room temperature are included in the Supplementary Material.
