# Supplementary material for: Enhanced terahertz detection of multigate graphene nanostructures
Source: Nanophotonics. 2022 Jan 4;11(3):519–29. doi: 10.1515/nanoph-2021-0573 (PMC11501572; doi:10.1515/nanoph-2021-0573)
Supplement: Supplementary file 1 — Supplementary Material Details [file j_nanoph-2021-0573_suppl.pdf]

Supplementary Material:

Enhanced Terahertz Detection of Multigate Graphene Nanostructures.

J.A. Delgado-Notario[1*], W. Knap[1], V. Clericò[2], J. Salvador-Sanchez[2], J. Calvo-Gallego[2], T. Taniguchi[3], K. Watanabe[4], T. Otsuji[5], V.V. Popov[6], D.V. Fateev[6], E. Diez[2], J.E. Velázquez-Pérez[2] and Y.M. Meziani[2]

[1]*CENTERA Laboratories, Institute of High Pressure Physics, Polish Academy of Sciences, 29/37 Sokołowska Str, Warsaw, Poland*

[2]*Nanotechnology Group, USAL-Nanolab, Universidad de Salamanca, Plaza de la Merced, Edificio Trilingüe, 37008, Salamanca, Spain*

[3]*International Center for Materials Nanoarchitectonics, National Institute for Materials Science, 1-1 Namiki, Tsukuba 305-0044, Japan*

[4]*Research Center for Functional Materials, National Institute for Materials Science, 1-1 Namiki, Tsukuba 305-0044, Japan*

[5]*Research Institute of Electrical Communication, Tohoku University, Sendai 980-8577, Japan*

[6]*Kotelnikov Institute of Radio Engineering and Electronics (Saratov Branch), Russian Academy of Sciences, Saratov 410019, Russia*

*email: jnotario@mail.unipress.waw.pl

SM1: Graphene based heterostructure

The fabrication of the graphene-based heterostructure starts with the exfoliation of a monolayer graphene sheet (Figure SM1 (a)). Relatively thick h-BN flakes were used as top (Figure SM1 (b)) and bottom (Figure SM1 (c)) dielectric layers and a few-layers graphite flake (Figure SM1 (d)). They were exfoliated on a SiO_2/Si substrate (300 nm SiO2) by micromechanical cleavage of bulk materials using an adhesive tape [1] and individually identified under an optical microscope. Thick h-BN flakes were also characterized by a Stylus Profilometers to estimate their thickness. Later, the graphene-based heterostructure was fabricated by using a polymer based dry transfer technique [2]. First, the top hBN flake (~28 nm) was transferred onto the graphene sheet and then, the hBN/Graphene heterostructure was cleaned to remove the polycarbonate film by rinsing in chloroform for a few minutes. Similarly, the process was repeated and the bottom hBN flake (~50 nm) was transferred onto the few-layers Graphite flake previously exfoliated on a SiO_2 substrate that will be used as the back-gate of the transistor. The graphite back-gate helps to screen the remote charge impurities trapped in the SiO_2 substrate and therefore could increase the quality and channel mobility of the final device [3]

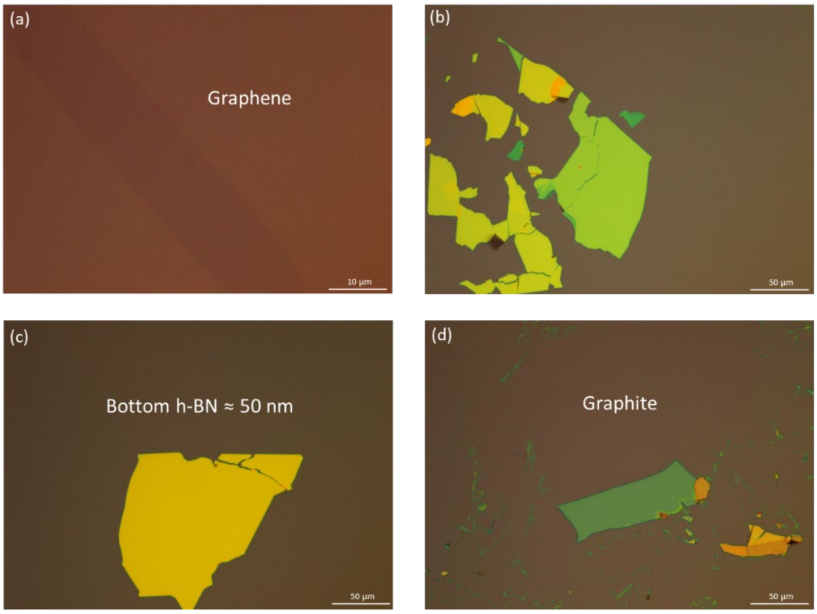

Figure SM1. Optical images of the exfoliated 2D materials. (a) Graphene, (b) top and (c) bottom h-BN and (d) graphite flakes exfoliated on a SiO_2/Si (300 nm of SiO_2) substrate before the staking.

The resulting van der Waals vertical heterostructure (hBN/Graphene/hBN/Graphite) was fabricated when the former hBN/Graphene heterostructure was picked up and transferred onto the hBN/Graphite heterostructure (Figure SM2 (a)). The final heterostructure was characterized by the Raman spectroscopy (micro-Raman spectrometer LabRAM HR Evolution at wavelength 532 nm and the incident power of ~1 mW) to identify and check the quality of the encapsulated graphene sheet (Figures SM2 (b) – (c)).

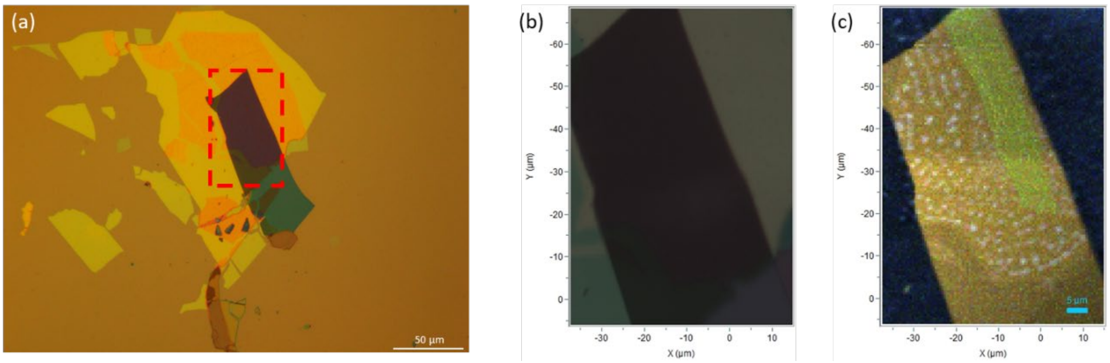

Figure SM2. (a) Optical image of the final heterostructure (hBN/Graphene/hBN/Graphite) fabricated, where the dashed red line shows the area characterized later by the Raman spectroscopy. (b) Optical zoomed image of the red dashed line area of the graphene-based heterostructure. (c) The Raman spectral mapping showing the graphene and graphite flakes highlighted in greenish and yellowish colors respectively.

The fabricated stack was patterned using electron beam lithography (EBL) to define Drain and Source contacts using a homemade PMMA (6% in chlorobenzene) as resist. Then the heterostructure was dry-etched in an ICP-RIE Plasma Pro Cobra 100 with a SF_6 atmosphere (40 SCCM, P = 6 mTorr, P = 75 W at 10 ºC) and the areas unprotected by a lithographic mask were removed to open the access to the graphene sheet [4]. Thereupon the sample was placed inside the e-beam evaporator where the one-dimensional Drain and Source metal contacts were made by evaporating at very low pressure (10-8 mbar) 3.5 nm of Cr (0.06 nm/s) and 60 nm of Au (0.25 nm/s). The etching recipe ensured a truncated square pyramid shape of the heterostructure with a contact angle of approximately 40º to the horizontal plane (Figure SM3 (a)-(b)). This helped to fabricate the evaporated contacts and ensured high-quality quasi one-dimensional ohmic contacts to the encapsulated graphene sheet. Finally, a second round of EBL using PMMA (4% in chlorobenzene) was used to fabricate the asymmetrically

situated top gates followed by electron beam evaporation of Cr/Au = 5/45 nm (Figure SM3 (c)).

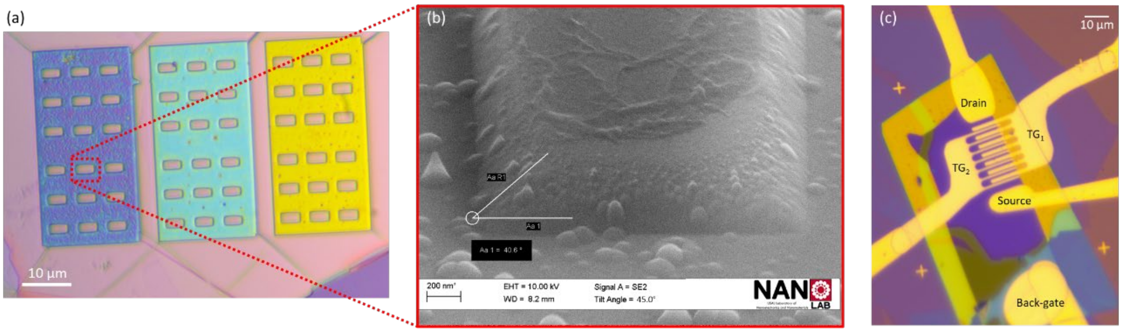

Figure SM3. (a) Optical image of a h-BN flake dry etched at 10ºC (blue region), –50ºC (cyan region) and –110ºC (yellow region) to define different rectangular patterns. (b) SEM tilted image of the area highlighted in the optical image showing the etching angle around 40º. (c) Optical photograph of the fabricated ADGG-GTeraFET where Drain, Source, Top Gate 1 (TG_1), Top Gate 2 (TG_2) and Graphite Back-Gate terminals are labeled.

SM2: Temperature dependence transport measurements

Figure SM4 (a) shows the experimental Drain-to-Source resistance (R_{DS}) as a function of the Back-Gate voltage (V_{BG}) at different temperatures. At 4.5K, an anomalous double peak on the R_{DS} curve was observed. This behavior was previously reported in multi-gated devices[5], [6] in which different doping profiles along the channel under the dual-gated and single-gated regions generated a bipolar junction when the Back-Gate bias voltage was in the range in which two maxima of the Drain to Source resistance took place. This observation can be explained as follows. Graphene sheet under each of the asymmetric top gates (TG_1 and TG_2) may exhibit a slightly different doping, which is also different from the doping in the single gated portion of the channel controlled only by the back-gate. In our device, a different doping between the dual gated and the single gated regions can appear for example due to a built-in voltage under the metallic top gates. The total sample resistance can be written as the following simple equation:

$$R_{DS} = R_1(BG) + R_2(BG, TG_1) + R_3(BG, TG_2) \quad (1)$$

where R_1 *(BG)* is the channel resistance of the single-gated regions of the channel that are controlled only by the graphite back-gate, while R_2 *(BG,*TG_1*)* and R_3 *(BG,*TG_2*)* are the contributions to the channel resistance of the dual-gated regions controlled by the top gates, TG_1 and TG_2 respectively, as well as by graphite back-gate. Equation 1 is a simple model of the channel resistance that depends on the different gates and neglects the aspects related to the interface between pads (Source and Drain) and the graphene channel (i.e. contact resistances) for the sake of simplicity. Here we assumed a slightly different doping under each of the top gates even if both gates were biased at the same voltage. Therefore, if dual- and single-gated regions have different values of charge neutrality point (CNP), the drain-to-source resistance of Equation 1 will look as in Figure SM4 (b). Firstly, for large negative back-gate bias voltages, the graphene sheet is non-uniformly p-doped. With the increase of the back-gate voltage, a very prominent first peak appears as the single-gated regions reach their CNP, while these dual-gated regions are still slightly p doped. At higher values of the back-gate voltage, a secondary peak comes out which corresponds to a situation in which the single-gated regions are slightly n doped but those dual-gated regions reach their CNP, and in consequence, different n-i junctions are created. Finally, at very large positive voltages, the channel is non-uniformly n-doped. This behaviour was only observed at low temperatures. With an increase of the temperature up to room temperature, a single and wider peak in the channel resistance was observed that could be understood as due to the blurring of the potential barriers with temperature.

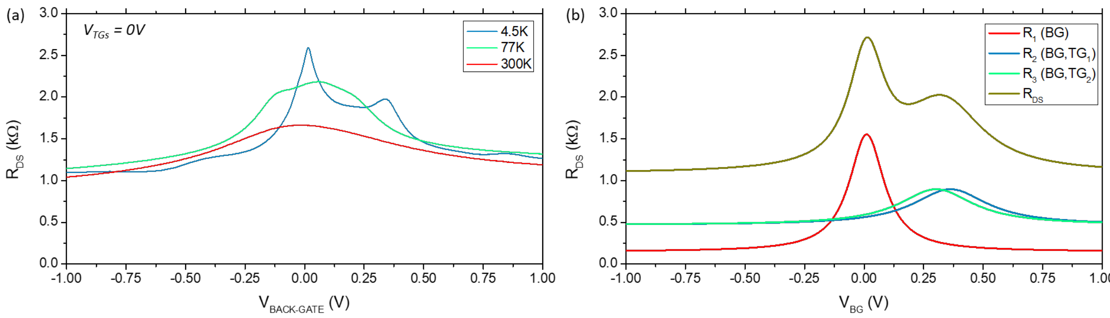

Figure SM4. (a) Experimental two-terminal resistance (R_{DS}) of the ADGG-GTeraFET as a function of the BG voltage at different temperatures and (b) modeled drain-to-source resistance curve of the ADGG-GTeraFET at 4.5K according to Equation 1 including those individual terms of the full resistance.

Figure SM5 shows the experimental Drain-to-Source resistance as a function of the TGs voltage when different BG voltages were applied at 77K (a) and at room temperature (b). Similar to the low temperature transport measurements at 4.5K, it was found that when different BG voltages were applied, the charge neutrality point (CNP) changed along with a vertical shift of the value of the channel resistance as shown in Figure SM5.

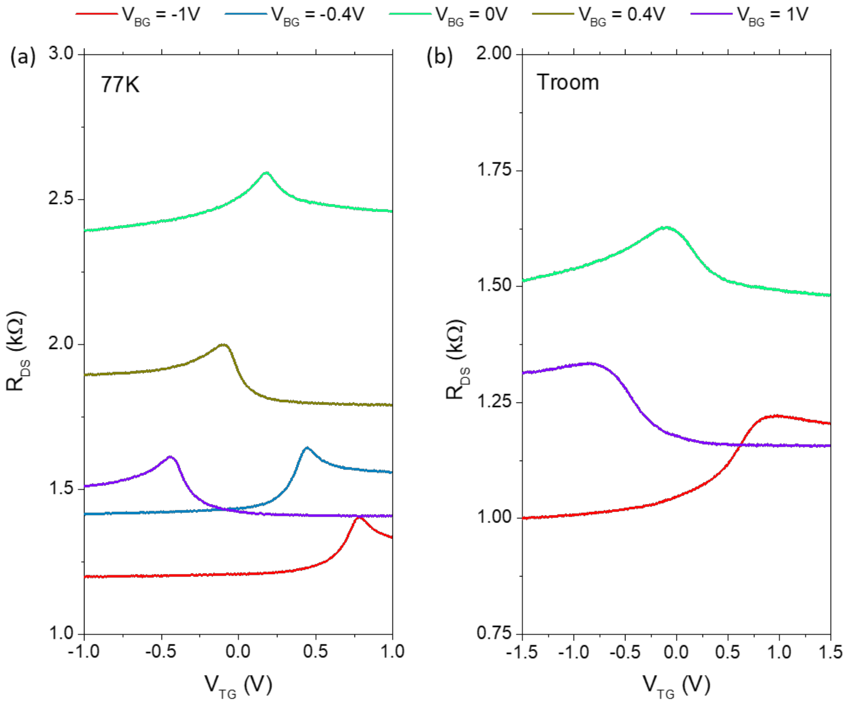

Figure SM5. Two-terminal resistance (R_{DS}) of the ADGG-GTFET as a function of the TGs voltage for different BG voltages at 77 K (a) and room temperature (b).

We noticed that a resistance change in the two-terminal configuration transport measurements was larger than a factor two while biasing with the graphite back-gate compared with biasing with the top-gates. The geometrical factor area between graphite and top gates is close to two which indicates that graphite back-gate has a better control over the carrier density in those dual gated regions that the metallic top gates when they were biasing simultaneously. Moreover, while biasing the graphite back-gate, the contact resistance may also be modulated, what cannot be achieved while biasing our top gates [7], [8]. Finally, the dual gate configuration in our device allows to create different lateral junctions along the graphene sheet while biasing the top gates and the back-gate. Nevertheless, the effective width of such a lateral junction in the graphene

sheet may be strongly affected either by the separation distance between metal and graphene itself or the permittivity of the material which may also led to a different resistance change when biasing either the top-gates or back-gates [9], [10].

SM3: Device characterization

From Drain-to-Source resistance, we can extract or estimate various key parameters of our device. Carrier mobility (μ_e for electrons and μ_h for holes), carrier concentrations and parasitic resistance (R_o) can be estimated [11], [12] since the Drain-to-Source resistance (R_{DS}) can be written as:

$$R_{DS} = R_o + \frac{N_{sq}}{n_{tot} e \mu} \tag{2}$$

where N_{sq} is the length-to-width ratio of the top-gated area, n_{tot} is the carrier concentrations (electrons or holes), e is the elementary positive charge and μ is the carrier mobility. Equation 2 gives the Drain-to-Source resistance as a serial resistance where the first term represents the sum of the contact resistances and the resistances of ungated graphene areas and the second term is the resistance of the gated regions of the graphene channel. Carrier concentration can be estimated using:

$$n_{tot} = \sqrt{n_o^2 + n^2} = \sqrt{n_o^2 + (\xi(V_G - V_{CNP}))^2} \tag{3}$$

where n_o is the carrier density at the CNP, n represents the carrier concentration induced by the gate, $\xi = \varepsilon_o \varepsilon_i / e d_i$ where ε_0 is the vacuum permittivity, and ε_i and d_i, are the relative permittivity and the thickness of the dielectric layer (i.e. h-BN in our device), respectively. Combining equations 2 and 3, the Drain-to-Source resistance can be written as

$$R_{DS} = R_o + \frac{N_{sq}}{\sqrt{n_o^2 + n^2} e\mu} = R_o + \frac{N_{sq}}{\sqrt{n_o^2 + (\xi(V_G - V_{CNP}))^2} e\mu} \quad (4)$$

Three different parameters, carrier mobility (μ), parasitic resistances (R_o) and either carrier concentration (n) or gate voltage (V_G) must be independently fitted, according to Eq. 4, to the experimental values of the drain-to-source resistance obtained by transport measurements in the range of 4.5K up to 300K and shown in Figure 2 in the manuscript and in SM2 section. These parameters must also be individually fitted in each branch to obtain the electron or hole carrier mobilities (μe and μh). Plots in Figure SM6 show two examples of the experimental (solid line) and the fitted (blue and red circles for electron and holes respectively) drain-to-source resistance curves obtained when varying the bias voltage of the back-gate, (a), and the Top Gates, (b), at two different temperatures. Experimental data of drain-to-source resistance can be fitted to as a function of the gate voltage (Figure SM6 (a)) or the carrier density (Figure SM6 (b)) according to equation (4). The experimental data are in excellent agreement with the ones fitted using equations (2)-(4) except in the case of RDS versus the back-gate at 4.5K in which the presence of two peaks in the transfer characteristics renders any fitting impossible.

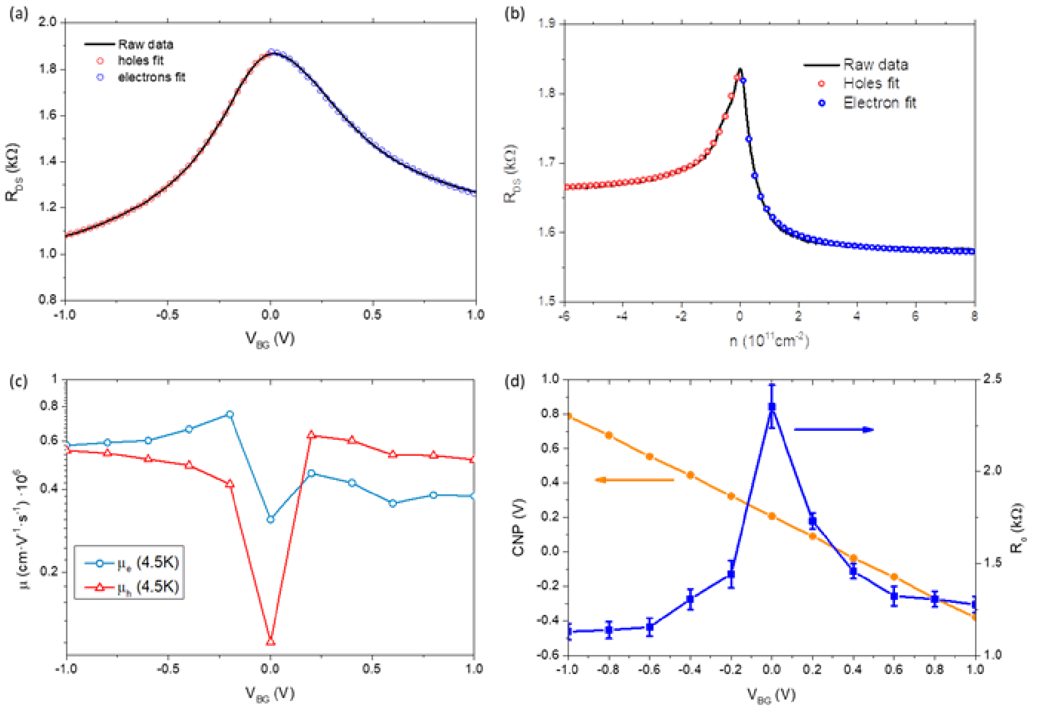

Figure SM6. (a) Experimental Drain-to-Source resistance (black line) as a function of the Back-gate voltage along with the fitting results (red and blue dots) at 300K. Top gates were kept at 0V. (b) Experimental Drain-to-Source resistance as a function of the Top Gates voltage along with the fitted results (red and blue dots) at 4.5K for a BG voltage of 0.4V. (c) Extracted hole and electron carrier mobilities and (c) the TGs voltage for the CNP and parasitic resistance as functions of the BG voltage at 4.5K.

The extracted values at 4.5K of the electron (and hole) mobility in the graphene channel of the transistor while biasing with the Top Gates were 78500 cm^{2}V^{-1}s^{-1} (and 74000 cm^{2}V^{-1}s^{-1}, respectively) (Figure SM6 (c)). When the BG is unbiased (V_{BG} = 0V), the graphene channel is close to the CNP and the extracted hole and electron mobilities are minimal, while the parasitic resistance exhibits its maximum value (Figures SM6 (c) and (d)). When the BG is biased at more positive (or negative) voltages, the new CNP is shifted towards more negative (or positive) TGs voltages, the parasitic resistance decreases (see Figure SM6 (d)) and the extracted mobility increases significantly (Figure SM6(c)). The extracted maximum values at room temperature of the electron (and hole) mobility in the device while biasing with the Top Gates were 38,000 cm^{2}V^{-1}s^{-1} (and 32000 cm^{2}V^{-1}s^{-1}). By comparison, the values of carrier mobility extracted by fitting analysis at room temperature while biasing with the back-gate were slightly better, with values around 40,500 cm^{2}V^{-1}s^{-1} and 38000 cm^{2}V^{-1}s^{-1} for electrons and holes respectively.

SM4: Experimental setup

Figure SM7 shows the schematic description of the experimental setup used for the THz photodetection experiments. The device was placed inside a variable temperature (4.5K – 300K) pulse-tube cryostat. A continuous wave of 0.3 THz radiation was generated by a solid-state harmonic source from RPG Radiometer Physics GmbH based on a dielectric resonator oscillator (DRO) oscillating at 12.5 GHz followed by Schottky diodes multiplier stages (multiplication factor of 24) with an output power of 6 mW. The output THz radiation was modulated by a mechanical chopper at 333 Hz, also providing a reference signal for the Lock-in Amplifier (Stanford SR860). The THz radiation was collimated by a 90º off-axis parabolic mirror (reflected focal distance = 15.25 mm) and finally focused onto the ADGG-GTeraFET by a TPX THz lens with a diameter of 50.8 mm and a focal length of 100 mm. The cryostat included an optical access based on a polyethylene window that is transparent to THz radiation. The photocurrent signal generated by the incoming THz radiation was collected at the Drain contact and the Source was kept grounded. In the experiments the signal was fed into a low-noise current-to-voltage preamplifier SR570 (amplification factor was set to 10^6 V/A) and the preamplifier output voltage was recorded by using a Lock-in Amplifier SR860.

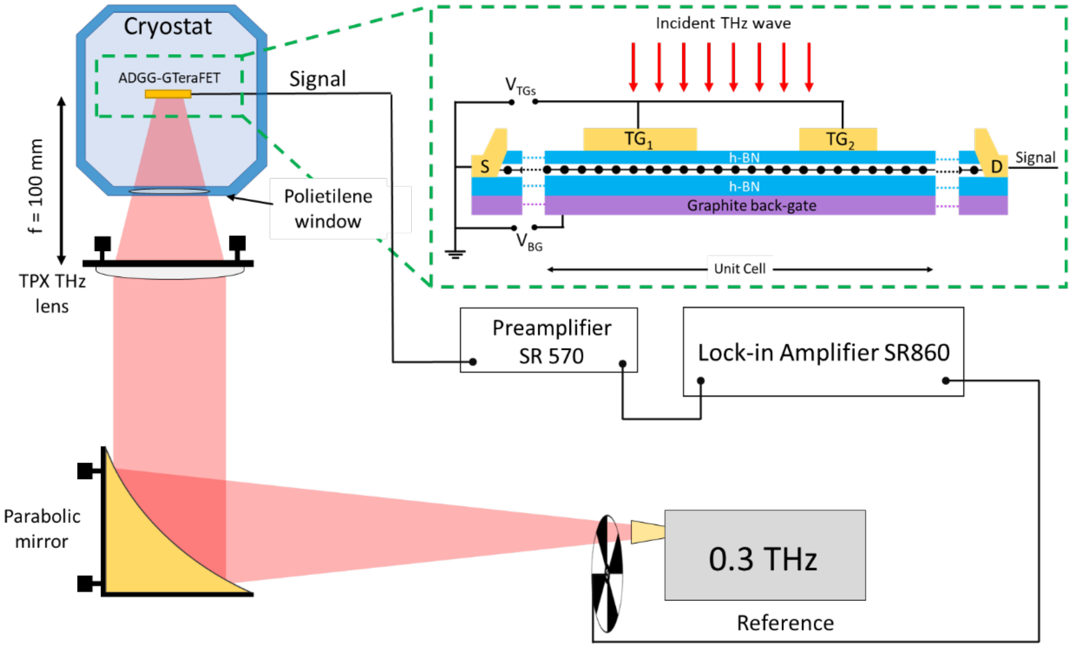

Figure SM7. Schematic view of the experimental setup for the THz detection experiments where the green dashed rectangle highlights the electrical configuration while detection measurements including only an asymmetric unit cell of the ADGG-GTeraFET for simplification.

SM5: Room temperature THz detection measurements

Figure SM8 shows the experimental THz photocurrent as a function of the TGs bias voltage for different positive (a) and negative (b) BG voltages under 0.3 THz illumination at room temperature. As shown in Figure SM8, the photocurrent is enhanced both on p and n sides around the CNP when positive or negative voltages are applied to the BG.

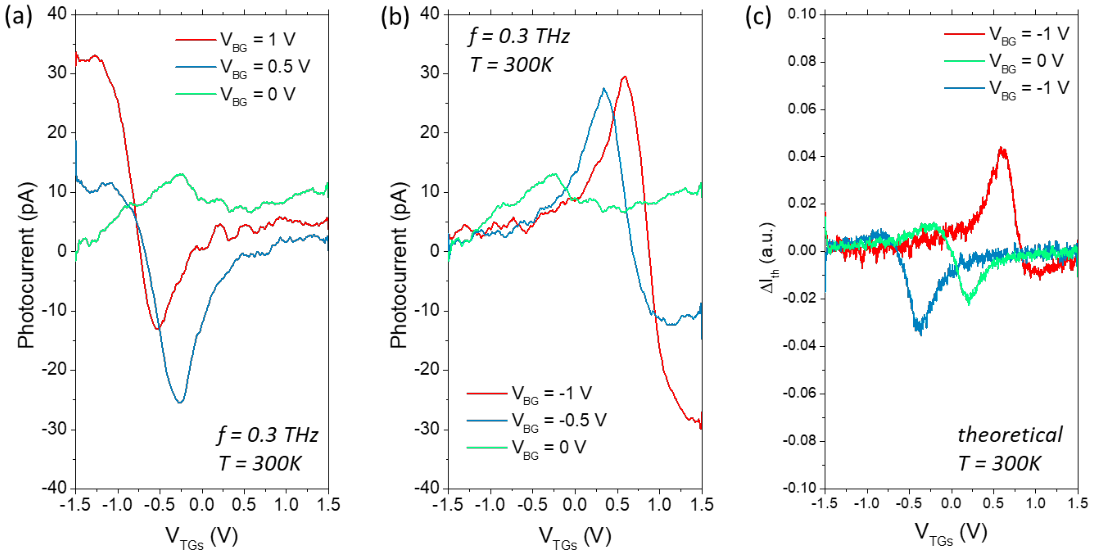

Figure SM8. Experimental THz photocurrent as a function of the TGs voltage for different positive (a) and negative (b) BG voltages under 0.3 THz illumination and theoretical photocurrent (c) at room temperature.

SM6. Photo-thermoelectric effect.

We performed additional experiments to verify that the Photo-thermoelectric (PTE) effect does not dominate in our ADGG-GTeraFET when “abrupt” potential barriers are created. Firstly, the photocurrent generated by the incoming THz radiation was measured while a source-to-drain bias voltage was applied to the device. Figure SM9 (a) shows the photoresponse measured as a function of the Top Gates (TGs) bias voltage for a back-gate bias of V_{BG} = -1V and for three different values of the source-to-drain bias voltages. It was found that the photoresponse can be modulated by changing the DC source-to-drain voltages. Also, the maximum value of the photoresponse shifted

towards a different value of TGs bias voltage when a source-to-drain bias voltage was applied as reported previously in other plasma-wave field-effect transistors[13].

Additionally, the photoresponse was measured when multiple "abrupt" p-n junctions were created along the graphene channel, i.e., for V_{BG} = -1V and V_{TGs} = 1V (orange dashed rectangle). Figure SM9 (b) shows the DC current (blue squares) and the photoresponse signal (red squares) when a DC source-to-drain voltage was applied in the ADGG-GTeraFET. Both DC current and the photoresponse under 0.3 THz excitation were measured simultaneously. The DC current grew linearly with the applied source-to-drain bias voltage, without any non-linearities. At the same time the photocurrent changed significantly with the source-to-drain bias voltages. These results are in contradiction with the main criteria of the identification of PTE as the dominant photocurrent mechanism as established in Ref. [14].

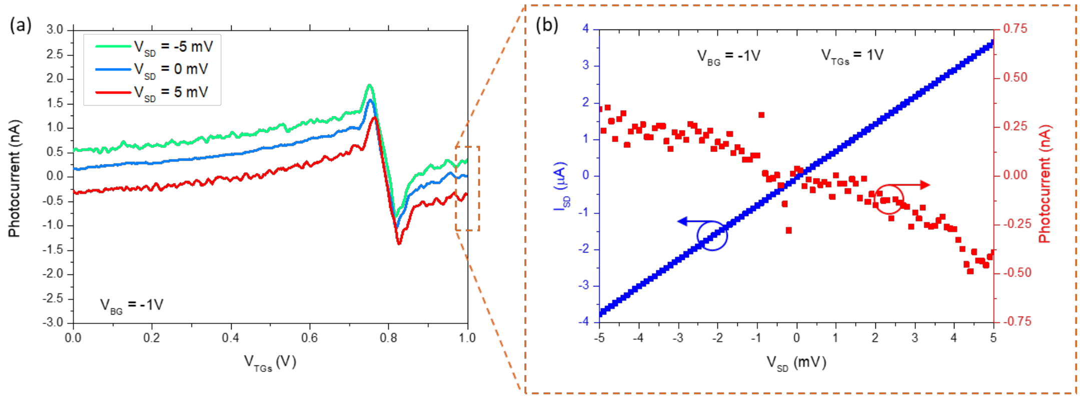

Figure SM9. Photoresponse signal as a function of the TGs voltage for a back-gate bias voltage of -1V. (b) Photocurrent and source-to-drain current measured as a function of the source-to-drain bias voltage. Incoming frequency was 0.3THz and T = 4.5K.

We also performed experiments when "smooth" potential barriers were created in the device when both top gates (TG_1 and TG_2) were independently biased. In contrast to the case of "abrupt" potential barriers, a considerably weaker signal was measured in the "smooth" potential barrier configuration. Moreover, no enhancement was observed in the "smooth" configuration. As an example, Figure SM10 (a) shows the photocurrent for four different gate bias polarizations. One can see that the results are totally superimposed one to another. Additionally, the photocurrent curves show two evident

sign changes while biasing with the TG_1. These results may suggest that observed photocurrent when "smooth" potential barriers are created could be dominated by the PTE effect.

When the temperature was increased up to 200K, the photocurrent mapping shows the 6-fold pattern characteristics for PTE effect (Figure SM10 (c)). Photocurrent mapping reveals different multiple sign changes that correspond to the transitions between the different carrier type configurations in the channel. These results are in agreement with previous works on graphene THz detectors exhibiting hot-carrier assisted PTE effect in which lateral junctions along the graphene sheet can be imposed by independent split gates [1zzf].

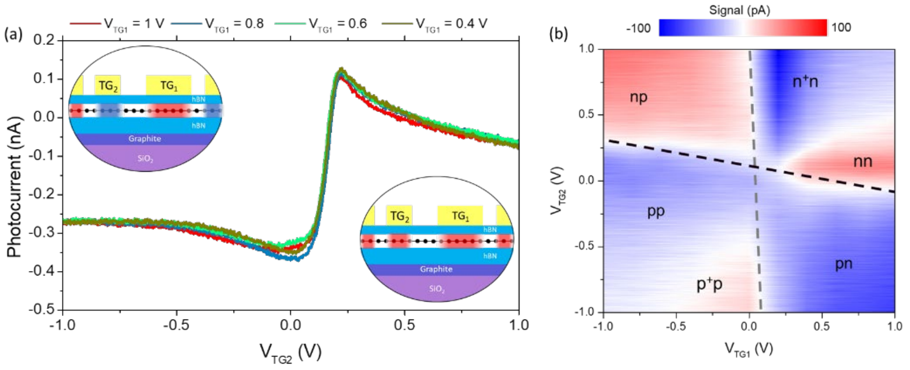

Figure SM10. (a) Photocurrent as a function of the TG_2 voltage for different positive TG_1 voltages at T = 77K. Inset figures show the polarization regimes at the asymmetric unit cell for two different bias regimes (p and n regions are plotted in blue and red colors respectively). (b) Photocurrent mapping under 0.3 THz radiation as a function of the voltage applied to both top gates at 200K.

References

[1] Y. Huang *et al.*, "Reliable Exfoliation of Large-Area High-Quality Flakes of Graphene and Other Two-Dimensional Materials," *ACS Nano*, vol. 9, no. 11, pp. 10612–10620, Nov. 2015.

[2] P. J. Zomer, M. H. D. Guimarães, J. C. Brant, N. Tombros, and B. J. van Wees, "Fast pick up technique for high quality heterostructures of bilayer graphene and hexagonal boron nitride," *Appl. Phys. Lett.*, vol. 105, no. 1, p. 013101, Jul. 2014.

[3] A. A. Zibrov *et al.*, "Tunable interacting composite fermion phases in a half-filled bilayer-graphene Landau level," *Nature*, vol. 549, no. 7672, pp. 360–364, Sep. 2017.

[4] L. Wang *et al.*, “One-dimensional electrical contact to a two-dimensional material.,” *Science*, vol. 342, no. 6158, pp. 614–7, Nov. 2013.

[5] J. A. Delgado-Notario *et al.*, “Asymmetric dual-grating gates graphene FET for detection of terahertz radiations,” *APL Photonics*, vol. 5, no. 6, p. 066102, Jun. 2020.

[6] J. Velasco, Y. Lee, L. Jing, G. Liu, W. Bao, and C. N. Lau, “Quantum transport in double-gated graphene devices,” *Solid State Commun.*, vol. 152, no. 15, pp. 1301–1305, Aug. 2012.

[7] S. M. Song, B. J. Cho, and A. Info, “Contact resistance in graphene channel transistors Review Articles,” *Carbon Lett.*, vol. 14, no. 3, pp. 162–170, 2013.

[8] F. Urban, G. Lupina, A. Grillo, N. Martucciello, and A. Di Bartolomeo, “Contact resistance and mobility in back-gate graphene transistors,” *Nano Express*, vol. 1, no. 1, p. 010001, Mar. 2020.

[9] F. A. Chaves, D. Jiménez, J. E. Santos, P. Bøggild, and J. M. Caridad, “Electrostatics of metal–graphene interfaces: sharp p–n junctions for electron-optical applications,” *Nanoscale*, vol. 11, no. 21, pp. 10273–10281, May 2019.

[10] Q. Wilmart *et al.*, “Contact gating at GHz frequency in graphene OPEN,” *Nat. Publ. Gr.*, 2016.

[11] S. Kim *et al.*, “Realization of a high mobility dual-gated graphene field-effect transistor with Al2O3 dielectric,” *Appl. Phys. Lett.*, vol. 94, no. 6, p. 062107, Feb. 2009.

[12] L. Gammelgaard *et al.*, “Graphene transport properties upon exposure to PMMA processing and heat treatments,” *2D Mater.*, vol. 1, no. 3, p. 035005, Nov. 2014.

[13] F. Teppe *et al.*, “Plasma wave resonant detection of femtosecond pulsed terahertz radiation by a nanometer field-effect transistor,” *Appl. Phys. Lett.*, vol. 87, no. 2, p. 022102, Jul. 2005.

[14] S. Castilla *et al.*, “Fast and Sensitive Terahertz Detection Using an Antenna-Integrated Graphene pn Junction,” *Nano Lett.*, vol. 19, no. 5, pp. 2765–2773, May 2019.

[15] L. Viti *et al.*, “Thermoelectric graphene photodetectors with sub-nanosecond response times at terahertz frequencies,” *Nanophotonics*, vol. 10, no. 1, pp. 89–98, Jan. 2020.

[16] A. Brenneis *et al.*, “THz-circuits driven by photo-thermoelectric, gate-tunable graphene-junctions,” *Sci. Reports 2016 61*, vol. 6, no. 1, pp. 1–9, Oct. 2016.
